# Country risk, global uncertainty, and firms’ cash holdings: Do the role of law, culture, and financial market development matter?

**DOI:** 10.1016/j.heliyon.2024.e26266

**Published:** 2024-02-20

**Authors:** Seyed Alireza Athari, Roland Cho Teneng, Bülent Çetinkaya, Mahboubeh Bahreini

**Affiliations:** a Department of Business Administration, Faculty of Economics and Administrative Sciences, Cyprus International University, Northern Cyprus, Turkey; b Department of Business Administration, European Leadership University, Northern Cyprus, Turkey

**Keywords:** Country risk, Global uncertainty, Cash holdings, Law, Culture, Financial market development, Asia

## Abstract

To the extent of our knowledge, there is a gap in scrutinizing the impacts of the country's risk and global uncertainty on Asian firms' cash holdings. Therefore, this work aims to shed light on this gap by choosing 989 listed non-financial Asian firms and performing both the static (fixed-effects) and dynamic (Difference-GMM and System‐GMM) panel data methods between 2008 and 2020. The findings reveal that an increase in a country's risk and global uncertainty stimulates firms to stockpile more cash though the impact of country risk is more pronounced. Likewise, the results underscore that among the country risk factors, firms have more precautionary motives to stockpile more cash with increases in financial and political instability while they hold more cash with rises in economic stability. Remarkably, the results emphasize that the impacts of country risk and global uncertainty on cash holdings are more prominent in environments characterized by common-law legal origin, high uncertainty avoidance cultures, and less financial market development. Moreover, the results reveal that firm-specific factors including growth opportunity, leverage, profitability, dividend, and size have an important role in shaping Asian firms' cash holdings policy.

## 1 Introduction

Cash is essential corporate liquid assets and companies prefer to stockpile greater amounts of cash and cash equivalents as a precautionary incentive to avoid foregoing future positive NPV projects [1] and preparing for future unpredicted contingencies and short-term debt crises [2]. Moreover, firms are probable to hold liquid assets as a transaction cost motive to avert the transaction costs of raising external financing and to fund profitable expansion opportunities [3]. However, stockpiling cash causes the carrying cost of the capital invested in liquid assets and also agency costs [4]. Consequently, this trade-off has necessary implications for maintaining the ideal level of cash reserves by corporate executives.

As firms stockpile considerable amounts of liquid assets, however, there is a substantial variation in cash holdings between companies operating in different countries. For instance Ref. [5], reported that the median cash holdings in the U.S. are US$ 19.5 million per firm, which is relatively higher than US$ 31.5 million in Switzerland [6]. found that the cash holdings of EMU (European Economic and Monetary Union) corporations were 15% in 2000 [7]. uncovered the average cash holdings ratio of U.S. industrial firms was 23.2 percent in 2006, which is more than double in 1980 [8]. showed that between 1989 and 2009, the percentage median cash holdings ratio was different across countries and varied from 2.3 for New Zealand to 16.6 for Hong Kong. Based on the information provided in the World Scope database, the average cash holdings ratio of firms in 45 economies has gradually increased, and it was over 15% between 1995 and 2004. The existence and increasing trends of cash holdings in firms around the world have attracted the interest of academic researchers and scholars.

How can we explain this substantial variation in firms' cash holdings policy behavior across countries? There is significant prior literature drawing from three important theories in corporate finance namely the trade-off, the pecking order, and the free cash flow theories to explain cash holdings. Over the past three decades, some empirical works have thoroughly examined the factors of cash holdings in financial and non-financial firms in advanced and emerging countries ([9,10]). Findings of previous works suggested that the factors of cash holdings decompose into the firm and country levels. Several works underscored that growth opportunity, leverage, profitability, dividend payouts, size, and corporate governance are significant firm-level factors ([[10], [11], [12]]) and country-level factors in particular macroeconomic factors (e.g., inflation, GDP growth) ([13,14]), financial development ([15,16]), and legal protection of investors ([17,18]) on cash holdings. Likewise, some works implied that the country's culture significantly impacted the cash holdings [7].

While extensive works are investigating the factors of cash holdings, few works have been conducted mainly to explore the impact of risk at both country and global levels on cash holdings. As discussed by ([17,19]), country policy uncertainty (e.g., fiscal, monetary, regulatory) is likely to impact the financial decisions of companies and a rise of uncertainty triggers the precautionary incentive of companies to hoard more cash to safeguard against financial shocks and unanticipated contingencies. Findings of prior empirical studies also suggested that firms by increases in macroeconomic and political risks are more likely to postpone investments and instead prefer to stockpile more cash as a precautionary and transaction motive to reduce the possibility of increases in external funding cost and the likelihood of financial distress ([12,14,18,20]). Moreover, several studies suggested that global policy uncertainty, in addition to country risk, impacts cash holdings. For instance Refs. [21,22], documented that a financial crisis positively impacted cash holdings, arguing that firms prefer to save more cash as a precautionary motive to safeguard against uncertainty in the crisis period [18]. showed that global political crises increase the market's perceived risk and cost of external funding for firms [9,23]. indicated that cash holdings are positively impacted by global policy uncertainty and firms prefer to hold more cash during worldwide uncertainty periods.

What is the cash holdings policy in Asian economies and which factors significantly impact cash holdings? In the descriptive analysis of Asian firms’ cash holdings, for instance Ref. [24], found that the average cash holdings ratio of firms operating in Malaysia, the Philippines, Indonesia, Singapore, and Thailand are 12.4% between 2001 and 2005 [25]. showed that the average cash holdings of firms in Taiwan were 13% between 1997 and 2009 [26]. revealed that firms in East Asian countries almost doubled their cash holdings ratio increasing from 6.7% in 1996 to 12.1% in 2006. Focusing on the empirical analysis, the results of previous research also confirmed that the above-mentioned traditional firm and country-level factors impact the cash-holding policies of Asian companies. For instance Ref. [27], found that the cash holdings of companies in Singapore and Malaysia are impacted by corporate governance, and companies with less efficient governance stockpile more cash than their counterparts.

As reviewed, Asian firms' cash holdings policy has not been extensively investigated from a risk perspective. Therefore, this work aims to shed light by answering the subsequent questions in the setting of Asian economies: i) How do country risk and global uncertainty impact cash holdings? ii) How do financial, economic, and political risks specifically impact cash holdings? iii) How do countries' characteristics matter in determining the nexus between country risk, global uncertainty, and cash holdings? This study contributes from several aspects. First, although a wide range of works explored the factors influencing cash holdings, few attempts have been made to focus on the nexus between country risk, global uncertainty, and cash holdings particularly in the context of Asian markets. Therefore, the present study contributes to conducting this relationship by selecting 989 publicly listed non-financial Asian companies and including the unique data set of the International Country Risk Guide (ICRG) composite country risk index. Second, this work makes another contribution to the literature by explaining how countries’ characteristics namely legal origin, culture, and financial market development impact the nexus between country risk, global uncertainty, and cash holdings.

This work provides some consistently remarkable highlights. First, the findings reveal that a rise in country risk and global uncertainty stimulates the precautionary incentive of companies to stockpile more cash though the impact of country risk is more prominent. This implies that policymakers and regulators in Asia by providing stable environments could reduce Asian firms' cash volatility and also discourage them from lowering liquidity. A rise in country risk drives companies to hoard more cash, leading to opportunity costs, inefficient capital allocation, missed investment opportunities, and eventually sluggish economic activities. Besides, the results suggest that by diversifying their assets portfolios, Asian corporate executives could reduce the spillover effect of global risk, and by holding lower cash can exploit investment opportunities, and ultimately increase yields. Second, the findings underscore that Asian firms have more precautionary motives to stockpile more cash with increases in financial and political instability while they hold more cash with rises in economic stability. Policymakers and regulators by controlling financial instability (e.g., decreasing exchange rate volatility) and political instability (e.g., decreasing corruption) could help Asian firms hold lower cash and instead exploit growth opportunities and increase firms' efficiency. Third, the findings reveal that countries’ characteristics matter, and the impacts of country risk and global uncertainty on cash holdings are more prominent in environments with common-law legal origin, high uncertainty avoidance cultures, and less financial market development characteristics.

The rest of this work is organized as follows. Section 2 describes cash holdings theory and reviews empirical studies. Section 3 discusses cash holdings determinants and hypothesis development. Section 4 explains the data and methodologies. Sections 5, 6 reveal the findings followed by robustness tests. Section 7 is the conclusion.

## 2 Corporate cash holdings: theory and empirical base

### 2.1 Cash holdings theories

Three prominent theoretical models, namely, the trade-off, the pecking order, and the free cash flow models, explain the cash holdings. According to the trade-off model, there are two major trade-off motives, namely the transaction cost and the precautionary motives. Companies can fund their financial deficits by reducing dividends, issuing new debt and new equity, and selling existing assets. Particularly, raising external funds has significant transaction costs. The transaction cost motive states that companies stockpile cash and liquid assets to meet expenditures associated with ongoing activities and possible unpredicted events to circumvent the transaction costs of raising external funds. The precautionary motive states that companies that have financial deficits are more likely to give up investing in profitable projects. These companies incur the cost because of the expected losses resulting from foregoing profitable investment opportunities. Moreover, companies with a more significant investment opportunity set are more likely to have higher financial concern costs because of the positive outcome (e.g., NPV) of these investments disappearing because of bankruptcy. In this regard, companies with greater investment opportunities accumulate higher cash to prevent financial distress ([28,29]).

Notably, the related costs are higher for companies with valuable investment opportunities since the transaction costs of raising external funding are higher due to the information asymmetries for such companies [3]. However, holding cash causes carrying costs. The trade-off model discusses that companies set an ideal level of cash holding when the marginal benefits of stockpiling cash are equal to the marginal costs. Unlike the trade-off model, the pecking order model suggests that there is no ideal level of cash holdings ([3,30]). Companies follow a financing hierarchy to alleviate the costs arising from the information asymmetry problem between insiders and outsiders. Companies try to minimize the asymmetric information costs by choosing internal financing first, and then debt financing, and equity financing as a last resort. Empirical works [31] showed that the pecking order theory can explain cash-holding decisions, especially in developing countries where the information asymmetry costs are relatively higher.

Within the framework of agency theory, the free cash flow model contributes to the understanding of cash holdings ([4,32]). Executives tend to accumulate excess cash for their benefit at the expense of shareholders and for having discretionary managerial power [4,33]. argued that managers by stockpiling more cash would enjoy higher discretionary power resulting in increasing agency conflicts. To alleviate agency problems, the free cash flow model argues that any excess cash flow should be distributed to shareholders [34]. explained that institutional factors, such as the rules of law, impact companies' financial decisions. According to the agency theory and the free cash flow model, a firm's cash holdings are affected by the effectiveness of governance mechanisms at the country level. In poor governance settings, agency costs are higher, external financing is costlier, and executives have more incentives to stockpile excess cash for their benefits ([35,36]).

### 2.2 Empirical evidence

The subject of cash holdings is widely investigated in the finance literature. For instance, focusing on firm-level cash holdings determinants [37], studied the factors of cash holdings of 90 thousand US companies. Their findings showed that, in general, companies with high growth opportunities and a high level of capital expenses tend to hold more cash. Also, their results highlighted that cash holdings are lower in large companies due to having more access to capital markets. Their empirical findings supported both the trade-off and the agency theories and showed that leverage and cash flow impact negatively and positively on cash levels, respectively.

[38] studied the impact of capital structure and dividend policy on cash holdings in some emerging economies and found significant evidence that dividend policy and capital structure affect cash holdings. The results of the cross-sectional analysis revealed that leverage negatively impacted cash holdings in BRIC countries, which suggested that companies with more access to debt financing are less in need of stockpiling cash. Also, a negative nexus between dividend payouts and cash holdings was reported except for Brazil and China, implying that the higher the access to capital markets causes the less need to stockpile cash. Moreover, a positive nexus between company size and cash holding is detected except for India, indicating large companies need to hoard more cash to diversify their opportunities. In another study [10], uncovered that cash reserves are smaller in companies with weaker corporate governance. The authors explained that in companies with a more ineffective governance system managers choose to devote cash to capital expenditures and acquisitions, rather than reserve it. In contrast [27], showed that companies with less effective governance systems prefer to save more cash relative to their counterparts [39]. corroborated the significant negative impact of corporate governance on cash holdings, indicating that firms stockpile less cash when they have strong corporate governance settings [40]. revealed that higher country-level governance quality is associated with higher capital ratios in Islamic banks [41]. uncovered that firms with stronger governance mechanisms hold less cash, and firm and country-level governance settings are complements.

Furthermore, the strand of literature has investigated to define the factors of cash holdings at the country level. In influential studies [5,17], showed that a significant part of the cross-country variation in cash holdings is described by country-specific features such as country risk, corruption, and shareholders’ rights. They discussed that high levels of country risk and corruption might increase the problem of agency costs for companies, particularly in countries with lower governmental, financial, and regulatory institutions qualities [17]. also reported a negative effect of inflation on cash holdings, indicating that companies tend to keep a lower amount of cash due to losing their value during inflation.

[5,38] showed that companies operating in low shareholder protection environments prefer to stockpile more amounts of cash, which supports the idea that shareholders cannot put pressure on managers to distribute cash. Similarly [6,18], argued that companies operating in environments with superior investor protection prefer to stockpile less cash. Consequently [42,43], discussed that the level of corporate risk-taking within a country is affected by formal institutions such as market development, investor protection, and the rule of law, which may result in changing cash levels [44]. showed that firms’ cash-holding policy is influenced by national culture, suggesting that in environments where people try to prevent uncertainty more, companies hold a more substantial amount of liquid assets.

Especially, in addition to the traditional cash holdings determinants at the corporate and country level, several studies have recently probed the impact of uncertainty on the cash holding decisions of companies. For instance Ref. [9], studied the impact of economic policy uncertainty (hereafter, EPU) on the cash-holding decisions of companies in the BRICS economies and found that companies have more tendency to stockpile more cash when EPU rises. They argued that the decision to stockpile cash could be considered a hedging instrument for companies against risk [46]. showed that during macroeconomic uncertainty (e.g., high inflation), managers face problems in predicting company-specific information, causing them to act more homogeneously.

However, the stable economic condition leads managers to act more idiosyncratically and enables them to decide more efficiently in allocating company resources, especially liquid assets. Consistently [8], mentioned that companies would boost their cash holding level when macro-economic uncertainty rises in the U.S [19]. also considered both the effects of governance settings and macro-economic uncertainty on cash holdings decisions and confirmed that uncertainty at the company and macro-economic level impact the levels of cash holding [23]. by probing the impact of global EPU corroborated that the risk increases the cautionary behavior of financially constrained companies, leading to hoarding more cash by decreasing both dividend payout and investment.

## 3 The determinants of cash holdings

### 3.1 Hypothesis development

Considering the cash holdings theories, this study hypothesizes the determinants’ signs. Table 1 displays the descriptions of using variables.

**Table 1:** Variables’ definitions and sources.

Variables	Definitions	Sources
*Dependent Variable*		
C/TA	Cash and cash balances to net assets ratio.	Worldscope
*Firm-level control variables*		
MtB	Growth opportunity is calculated as the market-to-book ratio.	Worldscope
TL/TA	Leverage is calculated as total liabilities over total assets.	
ROA	Profitability is calculated as net income over total assets.	
DIV	Dividend is calculated as dividends per share over earnings per share.	
Ln (TA)	Size is calculated as the natural logarithm of total assets.	
*Country and global-level control variables*		
DR	The annual country-level composite index score (0–100) of the International Country Risk Guide (ICRG) is used for measuring the country's risk level. ICRG uses a set of 22 components which are grouped into three subcategories financial, economic, and political risks. A high score means low country risks.	www.prsgroup.com
UAI	An uncertainty avoidance index is one of the cultural dimensions that expresses the extent to which the members of a society feel uncomfortable with an unknown future and uncertainty. A high score indicates that the country is less tolerant of ambiguity.	Hofstede (1980)
UAV	Uncertainty avoidance is the act of avoiding uncertainty by relying on established social norms, rituals, and bureaucratic practices. UAV refers to uncertainty avoidance value in society.	House et al. (2004)
LO	Country's legal origin. It equals 1 if the country has a common-law system and zero otherwise.	Treisman (2000)
IP	The annual score protecting minority investors (IP) (0–100) is used for measuring investor protection. IP score is measured by the extent of disclosure, director liability, ease of shareholder suits, shareholder rights, governance structure, and corporate transparency indices. A high score indicates that the country has a high level of investor protection.	www.doingbusiness.org
SMC	Stock market capitalization to GDP is measured by the total value of all listed shares in a stock market as a percentage of GDP.	Global Financial Development, www.twse.com.tw
SMT	Stock market turnover ratio is measured by the total value of shares traded during the period divided by the average market capitalization for the period.	Global Financial Development, www.twse.com.tw
GR	The annual global EPU index is used for measuring global uncertainty. The index is based on a GDP-weighted average of national EPU indices for 20 countries including Australia, Brazil, Canada, Chile, China, France, Germany, Greece, India, Ireland, Italy, Japan, Mexico, the Netherlands, Russia, South Korea, Spain, Sweden, the United Kingdom, and the United States.	www.policyuncertainty.com

Note: Table 1 describes the regression variables. The first and second columns provide the names and definitions of the variables that are employed in the econometric model.

Previous studies showed that companies prefer to stockpile more cash in riskier environments to buffer against increases in country risk, which resulted in increasing the possibility of cash volatility and shortage because of unpredicted cash flow deterioration ([17,46]). Consequently [47], stressed that the riskiness of cash flows triggers managers to stockpile more cash as a precautionary savings incentive in unstable countries [48]. revealed that companies with a higher level of exposure to macroeconomic uncertainty stockpile more cash and cash balances. Empirical studies ([14,17,18]) argued that the precautionary incentive of companies for holding liquid assets is more triggered by increases in financial and political risks because of the possible increased cost of external funding and financial shortfalls.

Likewise [49], found that companies during an economic recession prefer to stockpile more cash. The overall results showed a positive nexus between country uncertainty and cash holdings and underscored that managers might exercise cash holdings as an option in high-uncertainty periods to reduce the likelihood of financial distress from adverse cash flow shocks. However, several works ([13,50]) revealed that companies stockpile more cash and cash balances when countries have become economically stable. In this study, the country risk was measured by the country-risk composite index scores,^1^ and it is predicted to uncover a positive or negative significant nexus between country risk and cash holdings.

H1 There is a positive or negative significant nexus between country risk and cash holdings.

The nexus between global uncertainty and cash holdings have been investigated in several studies. For instance, the findings of [21,22] documented that a global financial crisis positively impacts cash holdings and argued that companies prefer to save more cash as a precautionary motive to shield them from uncertainty in the crisis period [18]. also showed that global political crises increase the market's perceived risk and cost of external funding for firms [9]. indicated that global EPU has a positive spillover impact on cash holdings, suggesting that companies stockpile more cash as an option to cope with uncertainty periods [23]. by constructing a global EPU index found that EPU leads to increased cash holdings, especially in financially constrained companies, as the result of the cautionary behavior of these firms. In this study, global uncertainty was measured by the annual global EPU index, and it is predicted to uncover a positive significant nexus between global uncertainty and cash holdings.

H2 There is a positive significant nexus between global uncertainty and cash holdings.

### 3.2 Control variables

#### 3.2.1 Firm-level control variables

Growth opportunity is a significant factor in determining a firm's cash holding. The trade-off and pecking order models argue that companies with profitable growth opportunities are more likely to stockpile cash ([12,18]). Nevertheless, the free cash flow theory expects a negative coefficient for growth opportunities [6]. argued that executives in companies with poor investment opportunities are more likely to accumulate cash and invest in unprofitable projects. Leverage is another significant factor in a firm's cash holding. According to the trade-off theory, on the one hand, companies that have easy access to debt financing may use borrowing as a substitute financing alternative, and they are predicted to stockpile less cash. On the other hand, managers of highly leveraged companies are more likely to stockpile more cash to alleviate the financial distress costs [51]. The pecking order model argues that leverage negatively impacts cash holdings. Companies use debt financing first when internal funds (e.g., cash and retained earnings) are insufficient. Using the free cash flow model [6], supported the negative effect of leverage and argued that leveraged firms hold less cash due to effective monitoring by lenders.

Profitability is also another significant determinant of a firm's cash holding ([5,11]). Following the pecking order model, profitable companies are expected to have more significant amounts of cash and retained earnings to decrease the cost of external funding and earnings volatility. Also, since cash is a result of funding and investment practices, therefore, profitable companies are better able to pay dividends, repay debts, and accumulate cash [5]. Furthermore, dividend payouts can be a significant factor that affects a firm's cash holding. Consistent with the trade-off theory, the nexus between dividend payouts and cash holdings should be negative. Companies that currently pay dividends may hold less cash because they can raise capital when needed more cheaply by reducing dividend payments than companies that do not pay dividends [38].

Moreover, size is a significant factor that negatively impacts a firm's cash holding. Large companies have less need to stockpile large amounts of cash because of have better access to capital and sources of funds at lower costs and are less prone to financial distress costs [38]. [52] argued that large firms stockpile less cash because of having less information asymmetry, resulting in lower costs of external financing. Several works supported the trade-off model and showed that size has a negative effect on cash holdings [12]. However, size also positively impacted cash holdings. According to the pecking order model, large companies are presumably more successful in generating cash flows, and hence, should have larger amounts of cash ([9,27]). Besides, according to the free cash flow model, executives of large companies are more likely to stockpile cash for using their benefits.

#### 3.2.2 Country-level control variables

Following the prior works, this study selects the country-level control variables, namely legal origins, culture, and financial market development. Numerous studies underscored that a country's governance quality (legal origin) [17], a country's culture [53], and financial market development [16] impact cash holdings. Based on the agency argument [4], companies prefer to stockpile less cash in economies with better legal systems (e.g., the common-law system). Likewise, the results of past works ([53,54]) confirmed that a country's culture impacts cash holdings, and firms prefer to stockpile more liquid assets in environments with higher uncertainty avoidance culture. Moreover, some studies highlighted that financial market development is a significant determinant, and companies operating in more developed financial markets have less transaction cost incentive to stockpile cash [15].

## 4 Data and Methodology

### 4.1 Data description

Initially, the sample of the present study consists of all publicly listed non-financial companies operating in Morgan Stanley Capital International (MSCI) All Country (AC) ASEAN Index between 2005 and 2021. However, the final sample of this study due to the unavailability of annual financial reports and missing observations, particularly for the country risk factor, was limited to only the 989 publicly listed non-financial companies operating in Indonesia, the Philippines, Malaysia, Singapore, Thailand, plus Taiwan countries during the 2008–2020 period. This study collects all annual data on firm-level financial variables from the Thomson Reuters WorldScope database.

**Fig. 1:**
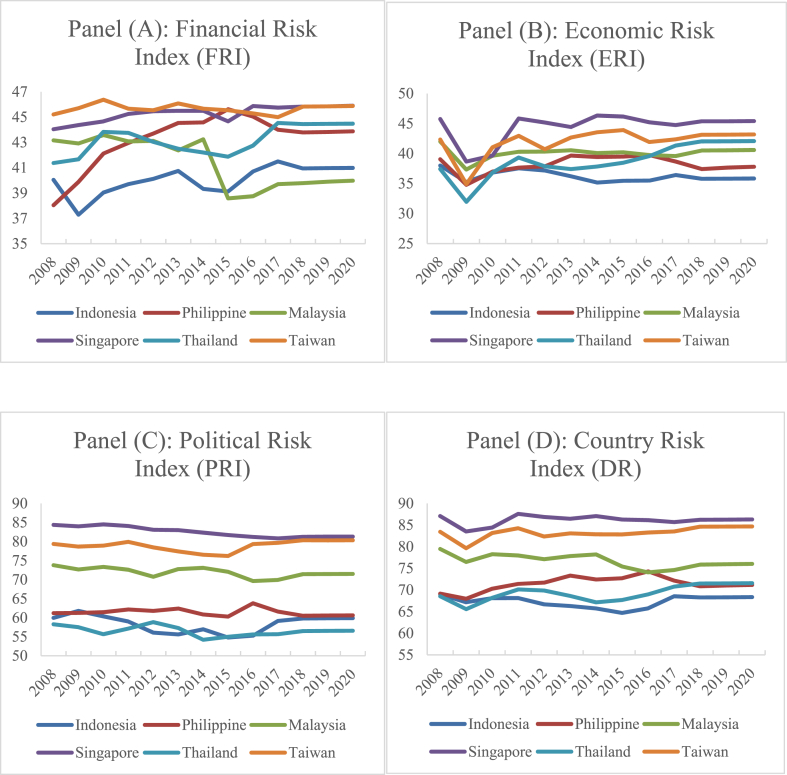
Time series plot of country risk index and its sub-indices for the sample countries.

Likewise, following the prior works ([[55], [56], [57]]), the annual composite index scores of the International Country Risk Guide (ICRG)^2^ were collected from the PRS Group to measure the country's risk. The ICRG uses a set of 22 components^3^ classified as the Financial Risk Index (FRI), Economic Risk Index (ERI), and Political Risk Index (PRI). As shown in Fig. 1 (Panels A, B, C, and D), ERI is more volatile relative to FRI and PRI, especially during the subprime mortgage crisis of 2008–2009.

Likewise, as suggested by Refs. [9,58], the annual global EPU index scores which are developed by Ref. [20] 4were collected for measuring global uncertainty. This study also follows prior studies ([7,53]), and the Uncertainty Avoidance Index (UAI) scores which were developed by Ref. [59] were collected for measuring the uncertainty avoidance culture. A high score denotes that the country is less tolerant of ambiguity. Besides, this study collected the uncertainty avoidance score, as in Ref. [54], for the robustness test. Moreover, this study uses the country's legal origin (LO) and investor protection (IP) as proxies for measuring institutional settings and external governance. In determining a country's LO, this study refers to Ref. [60] country classification, which grouped countries into the civil-law and common-law systems. For measuring IP, the annual scores of protecting minority investors were collected from the World Bank Group. This study also collected the annual data of the ratio of stock market capitalization to GDP (SMC), as in Ref. [53], and the stock market turnover ratio (SMT) from Global Financial Development for assessing financial development. Specifically, the annual data of SMC and SMT are hand-collected for the case of Taiwan from the Taiwan Stock Exchange website.^5^

Table A1 in the appendix presents the industry classifications and the number of firms in each industry. This study to control for the effect of each industry in the regression estimation uses the two-digit Industry Classification Benchmark (ICB) codes. As shown in Table A1, the majority of firms are concentrated in construction and materials (23.26%) and industrial engineering (18.08%). However, the industries of aerospace and defense (1.01%) and general industrials (5.06%) have the lowest share of firms. The sample firms include 49.31% of total firms in Indonesia, 84% in the Philippines, 73.18% in Malaysia, 84.80% in Singapore, 59.44% in Thailand, and 74.21% in Taiwan. Overall, the total sample firms contain 72.88% of the total firms in the examined sample countries.

Table A2 in the appendix reveals that there is no severe multicollinearity between the variables except between country-specific risks. The VIF also indicates that multicollinearity is not a serious problem.

### 4.2 Models and methodology

Eq. (1) performs to test the effects of country risk and global uncertainty on cash holdings by considering the firm and country-level factors.

Besides, Eq. (2) is used to particularly examine the impacts of country risk factors- FRI, ERI, and PRI- on cash holdings. Following [11], both equations include the year, industry, and country dummies but, for parsimony, the coefficients do not present it.

(1) $${C/TA}_{it\;}={\;\alpha }_{0}+\alpha_{1}{C/TA}_{it‐1}+{\;\alpha }_{2}\sum {Firm\;level\;control}_{it}+\alpha_{3}{Country\;risk}_{it}+{\;\alpha }_{4}\sum {Country\;level\;control}_{it}{+\alpha }_{5}{Global\;uncertainty}_{t}{+\varepsilon }_{it}$$

(2) $${\;C/TA}_{it\;}={\;\alpha }_{0}+\alpha_{1}{C/TA}_{it‐1}+{\;\alpha }_{2}\sum {Firm\;level\;control}_{it}+{Country\;risk\;factors}_{it}+{\;\alpha }_{4}\sum {Country\;level\;control}_{it}{+\alpha }_{5}{Global\;uncertainty}_{t}{+\varepsilon }_{it}$$

where _it_ represents firm and time, respectively. ε_it_ is an independent error term. C/TA is measured by cash and cash balances to net assets ratio. ${C/\text{TA}}_{\text{it}‐1}$ is the lagged cash holding; $\sum {Firm\;level\;control}_{it}$ includes growth opportunity (MtB), leverage (TL/TA), profitability (ROA), Dividend payout (DIV), and Size (Ln (TA)); country risk is the annual country-level composite index; $\sum {Country\;level\;control}_{it}$ includes legal origin (LO), uncertainty avoidance index (UAI), and financial market development (SMC); global uncertainty (GR) is the annual global EPU index.

After winsorizing factors, this study estimates the models by using the static and dynamic panel data methods. This work applies the static fixed effects panel data approach by clustering standard errors at the firm level. However, due to the presence of lagged dependent variables, we also employ dynamic panel data techniques to estimate more unbiased and consistent coefficients ([61,62]). In particular, performing dynamic panel data methods is more efficient when estimating models with endogeneity issues among explanatory variables. As discussed by Refs. [51,63], the inclusion of financial policies, capital structure, and dividend policy variables in the models may create endogeneity problems. Therefore, this work estimates the equations using the Difference-GMM [64] and System-GMM ([65,66]) methods to control the endogeneity problems. In probing the validity of the estimated models, the serial correlation, Hansen test, and also Sargan test were applied.

## 5 Univariate and multivariate analysis

### 5.1 Univariate results

Panel (A) of Table 2 shows the descriptive summary for each country. As shown in Panel (A), the sample includes six countries that comprise 989 publicly listed non-financial companies. Panel (A) indicates that the Philippines with 21 firms and Taiwan with 472 firms have the lowest and highest number of firms, respectively. Likewise, the descriptive summary reveals that Indonesia and Thailand, with the mean score of 67.308 and 69.231 for DR, are the most vulnerable countries, whereas, Taiwan and Singapore with the mean score of 80.496 and 86.144 are the least vulnerable countries, respectively. Besides, it reveals that Singapore, with a mean value of 8.000 for UAI and 4.220 for UAV, has the lowest level of uncertainty avoidance culture compared with other sample countries. Panel (A) also shows that Malaysia, Singapore, and Thailand have the common-law system while Indonesia, the Philippines, and Taiwan have the civil-law system.

**Table 2:** Descriptive statistics (2008–2020).

Panel (A): Average values of the variables for the investigated country															
Country	Total	Sample	C/TA	DR	UAI	UAV	LO	IP	% SMC	% SMT	MtB	TL/TA	ROA	DIV	Ln (TA)
Indonesia	73	36	0.112	67.308	48.000	5.230	0.000	58.334	39.529	30.686	1.685	0.534	0.032	0.191	21.091
Philippine	25	21	0.139	71.414	44.000	5.140	0.000	41.998	71.105	17.060	3.024	0.339	0.067	0.148	15.044
Malaysia	276	202	0.122	76.714	36.000	4.880	1.000	84.002	131.459	30.794	0.993	0.403	0.024	0.163	12.382
Singapore	204	173	0.128	86.144	8.000	4.220	1.000	87.998	226.579	41.423	1.237	0.451	0.012	0.192	11.979
Thailand	143	85	0.117	69.231	64.000	5.610	1.000	71.003	81.292	79.936	1.756	0.463	0.041	0.310	14.926
Taiwan	636	472	0.133	80.496	69.000	5.310	0.000	62.330	152.150	107.139	1.441	0.422	0.029	0.395	15.152
Total	1357	989	0.122	80.166	49.865	5.051	0.465	71.416	149.033	73.017	1.380	0.429	0.027	0.291	14.226

Panel (B): Descriptive statistics for the whole sample						
Variables	No. of. Obs.	Mean	Median	Std. Dev.	Minimum	Maximum
C/TA	12857	0.122	0.125	0.204	0.001	0.188
DR	12857	80.166	82.875	5.747	64.688	87.583
UAI	12857	49.865	64.000	23.209	8.000	69.000
UAV	12857	5.051	5.310	0.432	4.220	5.610
LO	12857	0.465	0.000	0.499	0.000	1.000
IP	12857	71.416	73.33	14.135	40.000	93.330
% SMC	12857	149.033	148.908	49.497	28.216	247.285
% SMT	12857	73.017	68.856	43.430	13.122	182.523
GR	12857	135.550	121.861	28.063	102.141	189.059
MtB	12857	1.380	1.000	1.584	−1.520	25.000
LEV	12857	0.429	0.424	0.211	0.001	2.786
ROA	12857	0.027	0.031	9.637	−0.934	0.639
DIV	12857	0.291	0.201	3.284	0.000	0.997
Ln (TA)	12857	14.226	14.245	2.423	5.858	24.624

Notes: Table 2 shows the descriptive values of the variables. For the country-specific, Panel (A) shows the number of firms and reports the mean values of each variable. Panel (B) provides a descriptive summary of the variables for all examined countries between 2008 and 2020. C/TA is the ratio of cash and cash balances to net assets; DR is the composite index of ICRG; UAI is the uncertainty avoidance index; UAV is the uncertainty avoidance value; LO is the country's legal origin which is equal to one when a country has the common-law system; IP is the protecting minority investor; SMC is the ratio of stock market capitalization to GDP; SMT is the stock market turnover ratio; GR is the global EPU index; MtB is market-to-book ratio; TL/TA is the ratio of total liabilities to total assets; ROA is the ratio of net income to total assets; DIV is dividend payout ratio; Ln (TA) is the natural logarithm of total assets.

Consequently, the descriptive statistics show in the common-law countries, IP is higher, and Singapore, Malaysia, and Thailand with a mean of 87.998, 84.002, and 71.003 have the highest IP scores. In contrast, IP is lower in the civil-law countries, and the Philippines, Indonesia, and Taiwan, with mean of 41.998, 58.334, and 62.330 have the lowest IP scores, respectively. Likewise, Panel (A) shows that Singapore, with a mean of 226.579 for SMC, and Taiwan with a mean of 107.139 for SMT, have the most developed financial markets. Panel (A) also shows that Indonesia and the Philippines with the mean of 0.112 and 0.139 have the lowest and highest cash holdings ratio (C/TA), correspondingly. Also, the Philippines has the highest mean value of growth opportunity (MtB) and profitability (ROA) with 3.024 and 0.067, respectively. For the leverage (TL/TA) and size (Ln (TA)) control variables, Indonesia has the highest value with a mean of 0.534 and 21.091, while Taiwan has the highest mean value of dividend with 0.395.

Panel (B) of Table 2 illustrates the descriptive summary for the entire sample. Panel (B) reveals that the median score of DR is 82.875, specifying that the whole sample economies are placed at a very low level of country risk. Besides, the median of 121.861 global uncertainty implies a high level of EPU at the global level. The descriptive statistics presented in Panel (B) also reveal that the median score of IP is 73.33 indicating a high level of shareholders’ rights in the whole sample countries. For the firm-level variables, the descriptive summary reveals that the median of C/TA is 12.20%. In addition, the median of MtB, TL/TA, ROA, DIV, and Ln (TA) variables are 1.000, 0.424, 0.031, 0.201, and 14.245, respectively.

Table 3 reveals specifically the descriptive statistics of country risk factors. The descriptive summary reveals that Indonesia, with a median score of 40.125 for FRI, is the most vulnerable country to financial risk followed by Malaysia and Thailand with a median score of 42.375 and 43.042, respectively. Likewise, Indonesia, with a median score of 36.840, and Singapore with a median score of 45.375, have the highest and lowest ERI levels, correspondingly. Moreover, the descriptive summary highlights that Thailand, with a median score of 56.550 for PRI, is the most vulnerable country to political risk, whereas Singapore, with a median score of 82.333, is the least vulnerable country. Overall, Table 3 shows that the whole sample countries with a median score of 45.208 for FRI and 41.333 for ERI are placed at a very low level of financial and economic risk. In contrast, it is placed at a low level of political risk by having a median score of 78.458 for PRI.

**Table 3:** Descriptive summary of country risk factors (2008–2020).

Country	Financial Risk Index (FRI) (0–50)			Economic Risk Index (ERI) (0–50)			Political Risk Index (PRI) (0–100)		
	Median	Minimum	Maximum	Median	Minimum	Maximum	Median	Minimum	Maximum
Indonesia	40.125	37.291	41.500	36.840	35.166	38.000	59.166	54.792	61.792
Philippine	43.830	38.042	45.625	37.875	34.792	39.750	61.250	60.292	63.792
Malaysia	42.375	38.583	43.583	40.291	37.333	42.000	72.042	69.625	73.792
Singapore	45.510	44.042	45.875	45.375	38.667	46.333	82.333	80.833	84.500
Thailand	43.042	41.375	44.542	38.458	31.958	41.333	56.550	54.208	58.833
Taiwan	45.667	45.000	46.375	42.645	34.917	43.917	78.916	76.208	79.917
Total	45.208	37.292	46.375	41.333	31.958	46.333	78.458	54.208	84.500

Note: Table 3 shows the descriptive statistics of country risk factors namely financial, economic, and political risks.

### 5.2 Multivariate results

Table 4 displays the findings of Eq. (1) using the fixed effects, Difference-GMM, and System-GMM methods for the entire countries. Table 4 shows that the coefficient of the lagged cash (C/TA) is positive and statistically significant, indicating that the Asian companies are partially adjusting their cash levels to meet target cash. Likewise, the results reveal that growth opportunity (MtB) has a positive effect on cash holdings. The positive relationship supports the trade-off and the pecking order theories and also previous works ([18,36]), indicating that companies stockpile more cash as a precautionary motive when profitable investment opportunities exist. Besides, the estimation results reveal that leverage (TL/TA) has a negative and significant impact on cash holdings. The negative effect supports the substitute impact of debt financing, the pecking order, the free cash flow theories, and also prior works [36].

**Table 4:** The impacts of the country risk and global uncertainty on cash holdings.

Independent variables	Dependent variable: C/TA		
	Fixed Effects (FE)	(Diff-GMM)	(Sys-GMM)
Lagged C/TA	0.355*	0.374*	0.336*
	(4.28)	(5.25)	(3.57)
*Firm-level variables*			
MtB	0.007	0.08**	0.005
	(1.25)	(2.16)	(0.79)
TL/TA	−0.278*	−0.153*	−0.244**
	(-5.81)	(-4.37)	(-2.06)
ROA	0.005**	0.001	0.023***
	(2.03)	(1.34)	(1.68)
DIV	0.002*	0.002	0.003**
	(3.06)	(0.78)	(2.06)
Ln (TA)	−0.024*	−0.033**	−0.032**
	(-2.72)	(-2.14)	(-2.12)
*Country and global-level variables*			
DR	−0.158***	−0.114*	−0.162*
	(-1.68)	(-4.58)	(-4.56)
LO	−0.014*	−0.009***	−0.007
	(-3.47)	(-1.71)	(-1.55)
UAI	0.021	0.017	0.025**
	(0.78)	(1.47)	(2.02)
SMC	−0.126	−0.096**	−0.132***
	(-1.43)	(-2.12)	(-1.68)
GR	0.051*	0.047***	0.035**
	(6.49)	(1.72)	(2.07)
Constant	0.244*	−0.427	0.732**
	(2.82)	(-1.15)	(2.11)
Y/I/C dummies	Yes	Yes	Yes
F-statistic	75.38*	–	–
Adj.R^2^	0.57	–	–
AR (2)	–	(0.323)	(0.311)
Hansen-test	–	(0.341)	(0.226)
Sargan-test	–	(0.225)	(0.332)

Note: This table shows the determinants of cash holdings for the whole sample by employing the Fixed Effects, Difference-GMM, and System-GMM approaches between 2008 and 2020. C/TA is the ratio of cash and cash balances to net assets; MtB is the market-to-book ratio; TL/TA is the ratio of total liabilities to total assets; ROA is the ratio of net income to total assets; DIV is dividend payout ratio; Ln (TA) is the natural logarithm of total assets; DR is the country risk index; LO is the country's legal origin; UAI is the uncertainty avoidance index; SMC is the Stock market capitalization to GDP ratio; and GR is the global EPU index. Dummies include the years, industries, and countries. For coefficients in the models, t-statistics are reported in parentheses. The symbols *, **, and *** indicate statistical significance at the 1%, 5%, and 10% levels, respectively.

Table 4 also reveals that profitability (ROA) has a positive and significant impact on cash holdings. This finding supports the pecking order theory and the prior works ([9,51]), implying that profitable companies stockpile a greater amount of cash as a precautionary incentive to diminish the external financing costs, and earnings volatility, and avoid missing profitable investment opportunities. Besides, Table 4 highlights that DIV has a significant and positive impact on cash holdings. As discussed by Ref. [52], companies that pay dividends are more likely to stockpile more cash as a precautionary saving motive to pay out dividends smoothly. Also [51], argued that companies operating in the less developed capital market have more tendency to stockpile a higher level of cash to pay smooth dividends to their shareholders and keep their reputation among investors. Moreover, the findings uncover that the size (Ln (TA)) has a negative and significant impact on cash holdings, supporting the trade-off theory. Previous works [18] suggested that large companies need less cash because of economies of scale in cash management and easier access to external financing.

Furthermore, Table 4 reveals that the coefficient of DR is negative and significant, denoting that Asian firms stockpile more cash by increasing the countries' vulnerabilities. Supporting the trade-off theory and the prior works ([18,48]), firms operating in more vulnerable environments prefer to stockpile more cash as a hedging instrument to have a safe shield against any potential adverse shocks and avoid financial distress. Likewise, some works ([63,67,68]) argued that companies operating in more vulnerable environments have more of a precautionary savings incentive to hold greater amounts of cash to protect against the higher possibility of cash volatility and shortage because of unpredicted cash flow deterioration, which resulted in forging some profitable investment projects. Besides, as we expected, Table 4 reveals that the coefficient of GR is positive and significant [45]. stated that managers might exercise cash holdings as an option in high uncertainty periods, and companies increase their liquid assets when there is a rise in uncertainty level. In line with [9], global EPU has a significant spillover effect on the firm's capital decisions of firms and companies stockpile more cash by rising in global EPU [50]. also stressed that firms during policy uncertainty stockpile more cash as a precautionary incentive to decrease the likelihood of financial distress. Remarkably, the results highlight that both DR and GR significantly affect cash holdings though the degree of the impact of DR has been more pronounced.

Moreover, the findings in Table 4 uncover that the coefficient of legal origin (LO) is negative and significant, indicating that firms stockpile less cash in countries with better legal systems (e.g., the common-law system) ([4,17]). Likewise, Table 4 highlights that uncertainty avoidance (UAI) with a positive sign and financial market development (SMC) with a negative sign significantly impact Asian firms’ cash holdings, respectively. This supports past works [7], in which findings reveal that companies stockpile more cash in environments with higher uncertainty avoidance culture. Also, it supports the prior works ([16,18]) which showed that the financial market development significantly impacted cash holdings and firms have less transaction cost incentive to stockpile cash in developed financial markets environments.

Table 5 reveals the findings of Eq. (2) by using the fixed effects, Difference-GMM, and System-GMM methods for the entire countries. Specifically, Table 5 shows the effects of country risk factors on cash holdings. The results reveal that the coefficients of FRI and PRI are negative and statistically significant while the coefficient of ERI is positive and significant. This implies that companies stockpile more cash by increasing the countries' vulnerability to financial and political risks though firms hold more cash by increasing countries’ economic stability.

**Table 5:** The effects of country risk factors on cash holdings.

Independent variables	Fixed Effects (FE)	(Diff-GMM)	(Sys-GMM)
	Panel (A): Financial Risk Index (FRI)		
Lagged C/TA	0.327**	0.248*	0.311*
	(2.18)	(4.48)	(5.18)
DR	−0.112**	−0.104**	−0.119*
	(-2.12)	(-2.04)	(-3.36)
Firm, country, & global control variables	Yes	Yes	Yes
Y/I/C dummies	Yes	Yes	Yes
F-statistic	65.88*	–	–
Adj.R^2^	0.43	–	–
AR (2)	–	(0.235)	(0.319)
Hansen-test	–	(0.426)	(0.556)
Sargan-test	–	(0.377)	(0.413)
	Panel (B): Economic Risk Index (ERI)		
Lagged C/TA	0.265*	0.277**	0.332***
	(3.44)	(2.11)	(1.73)
DR	0.093*	0.087***	0.114***
	(4.61)	(1.68)	(1.74)
Firm, country, & global control variables	Yes	Yes	Yes
Y/I/C dummies	Yes	Yes	Yes
F-statistic	65.42*	–	–
Adj.R^2^	0.52	–	–
AR (2)	–	(0.256)	(0.257)
Hansen-test	–	(0.417)	(0.442)
Sargan-test	–	(0.328)	(0.515)
	Panel (C): Political Risk Index (PRI)		
Lagged C/TA	0.324***	0.249*	0.287*
	(1.78)	(5.16)	(4.74)
DR	−0.328*	−0.298***	−0.305**
	(-6.84)	(-1.68)	(-2.09)
Firm, country, & global control variables	Yes	Yes	Yes
Y/I/C dummies	Yes	Yes	Yes
F-statistic	63.02*	–	–
Adj.R^2^	0.47	–	–
AR (2)	–	(0.227)	(0.355)
Hansen-test	–	(0.328)	(0.226)
Sargan-test	–	(0.442)	(0.413)

Note: This table shows the effects of country risk factors on cash holdings. Firm-level control variables include: MtB is the market-to-book ratio; TL/TA is the ratio of total liabilities to total assets, ROA is the ratio of net income to total assets, DIV is dividend payout ratio, Ln (TA) is the natural logarithm of total assets. Country-level control variables include: LO is the country's legal origin, UAI is the uncertainty avoidance index, SMC is the financial market development, and GR is the global EPU index. Dummies include the years, industries, and countries. The symbols *, **, and *** indicate statistical significance at 1%, 5%, and 10% levels, respectively.

The studies by Refs. [14,17] argued that the precautionary incentive of companies for holding liquid assets is more triggered by increases in financial and political risks [18]. also stressed that during periods of high political risk, firms have more precautionary motives to stockpile larger cash to shield against financial distress and maintain smooth financial activities. However, firms react differently to the economic risk and prefer to hold more significant cash when countries have become more economically stable. Similarly, the findings of several studies indicated that companies stockpile more cash in environments with less vulnerable to economic risk by having higher GDP growth ([13,14]) and lower inflation rate [44]. Notably, the results highlight that among country risk factors, the extent of the impact of PRI has been more prominent and firms react more significantly to increasing PRI relative to FRI and ERI.

### 5.3 Further analysis: do country characteristics matter?

As displayed in Table 6, the results reveal that the impacts of DR and GR on cash holdings depend on the countries’ characteristics, and their impacts are more pronounced in countries with the common-law system, high uncertainty avoidance culture, and less financial market development. Several works provided significant support for the impact of governance factors [17], cultural value [7], and financial development [16] on corporate cash-holding decisions. In line with works by Refs. [43,53] which with compelling evidence suggested the negative relationship between legal settings (e.g., legal origin, creditor rights) and corporate risk-taking decisions, our results imply that companies operating in strong legal settings environments are more likely to stockpile larger cash and cash equivalent than using external financing (e.g., debt financing) in response to the increases of country and global risks. Also, as suggested by prior studies ([17,39,69]), companies in common-law countries due to holding smaller cash have a relatively more precautionary incentive to stockpile more cash as a safeguard against increases of country and global risks. This precautionary motive is even more exacerbated in common-law countries given the fact that the agency problems due to the higher investor protections are less pronounced, and well-protected shareholders are more likely to accept holding more cash to hedge as an instrument when country and global risks increase.

**Table 6:** The effects of country risk and global uncertainty on firms' cash holdings policy under the different countries’ characteristics using the Sys-GMM approach.

Independent variables	Legal Origin (LO)		Culture (UAI)		Financial Markets (SMC)	
	Civil law	Common law	Low uncertainty	High uncertainty	Less development	High development
L.C/TA	0.415*	0.346*	0.235**	0.366*	0.322**	0.451*
	(4.26)	(3.75)	(2.13)	(6.65)	(2.05)	(4.66)
DR	−0.121**	−0.391*	−0.124*	−0.426*	−0.236***	−0.154***
	(-2.05)	(-2.98)	(-5.99)	(-2.64)	(-1.92)	(-1.68)
GR	0.011*	0.028**	0.016**	0.024**	0.067*	0.027**
	(3.25)	(2.41)	(2.17)	(2.04)	(3.14)	(2.35)
Firm & country Control variables	Yes	Yes	Yes	Yes	Yes	Yes
Y/I/C dummies	Yes	Yes	Yes	Yes	Yes	Yes
AR (2)	(0.255)	(0.312)	(0.426)	(0.304)	(0.411)	(0.346)
Hansen-test	(0.332)	(0.348)	(0.247)	(0.424)	(0.414)	(0.238)
Sargan-test	(0.265)	(0.413)	(0.287)	(0.335)	(0.285)	(0.362)

Note: This table shows how the countries' characteristics impact the nexus between country risk, global uncertainty, and firms' cash holdings using the Sys-GMM approach. Firm-level control variables include: MtB is the market-to-book ratio; TL/TA is the ratio of total liabilities to total assets, ROA is the ratio of net income to total assets, DIV is dividend payout ratio, Ln (TA) is the natural logarithm of total assets. Country-level control variables include: LO is the country's legal origin, UAI is the uncertainty avoidance index, SMC is the financial market development, and GR is the global EPU index. Dummies include the years, industries, and countries. The symbols *, **, and *** indicate statistical significance at 1%, 5%, and 10% levels, respectively.

Besides, the results underscore that companies operating in environments with high uncertainty avoidance culture stockpile more considerable cash by increases in DR and GR. Our findings imply that the higher level of uncertainty avoidance culture is directly linked with holding larger precautionary cash as a hedging instrument against possible future uncertainties (e.g., cash flow volatility) and also possible cash shortfalls incurred by increases in country and global risks. In environments with a high uncertainty avoidance culture, people are relatively more risk-averse and have more incentive to hold safe assets. Also, the higher uncertainty avoidance culture impacts the precautionary demand of firms to save more cash as a compensation tool for bearing ambiguity. In a prominent study [70], showed that the precautionary incentive for saving is triggered by the level of tolerance for uncertainty, and individuals hold more considerable cash in a high uncertainty avoidance culture [59,71]. documented that individuals in high uncertainty avoidance cultures feel more anxious in ambiguous, surprising, or unstructured situations and hence are inclined to take instant action to decrease the uncertainty level. Consistently [7], confirmed that uncertainty avoidance has a negative impact on corporate risk-taking, and companies prefer to stockpile more liquid assets in environments with higher uncertainty avoidance culture.

Moreover, the findings uncover that the impacts of DR and GR are more prominent in economies with less financial market development, and firms hold more cash and cash by increases in DR and GR. Our results support the prior works [16], which findings showed that companies in environments with more developed financial markets have less transaction cost incentive to stockpile cash due to easier access to capital markets and lower external financing costs. Besides, the prior studies ([72,73]) argued that increases in policy uncertainty lead to increasing the cost of external funding and exacerbate financial constraints for firms, driving them to stockpile more considerable amounts of cash than those unrestricted firms.

## 6 Robustness checks

Some robustness analyses were performed in this study to check the reliability of the result. First, this study estimates Eqs. (1), (2) by using a different dependent variable of “LOG C/TA”^6^ as used in several prior works ([7,39]). Second, this study uses the new firm-level proxies of the “total debts to total assets ratio” (TD/TA) for measuring the leverage and “net income to total equities” (ROE) for calculating the profitability in the estimation of Eqs. (1), (2), respectively. Third, as a further robustness check, this study additionally uses the alternative proxies for the country-level control variables namely “minority investor protection” (IP) for measuring the quality of institutional settings, as in Ref. [44], the “uncertainty avoidance value” (UAV) for measuring the uncertainty avoidance culture, as in Ref. [54], and also the “stock market turnover ratio” (SMT) for measuring the financial market development, as in Ref. [14]. Fourth, following [51,63] suggestions, we estimate Eqs. (1), (2) by using GMM in-Sys while firm-level control variables are considered as both exogenous and endogenous. Table 7, Table 8 reveal the findings of the robustness tests.

**Table 7:** Robustness test I.

Independent variables	Dependent variable: C/TA		Dependent variable: Log C/TA	
	Sys-GMM (Exogenous)	GMM in-Sys (Endogenous)	Sys-GMM (Exogenous)	GMM in-Sys (Endogenous)
Lagged C/TA	0.446*	0.421*	0.395*	0.418*
	(5.36)	(4.47)	(3.38)	(5.42)
*Firm-level variables*				
MtB	0.003	0.05	0.008	0.012**
	(0.68)	(1.44)	(1.23)	(2.05)
TD/TA	−0.143***	−0.162**	−0.223*	−0.133**
	(-1.68)	(-2.18)	(-4.12)	(-2.09)
ROE	0.005*	0.002	0.012**	0.013*
	(4.23)	(0.67)	(2.03)	(3.56)
DIV	0.001	0.004**	0.001	0.005***
	(1.08)	(2.17)	(1.55)	(1.71)
Ln (TA)	−0.021*	−0.026***	−0.034*	−0.018
	(-4.22)	(-1.69)	(-5.47)	(-1.04)
*Country and global-level variables*				
DR	−0.147*	−0.126***	−0.153*	−0.144**
	(-5.36)	(-1.69)	(-3.75)	(-2.12)
IP	−0.011	−0.015**	−0.009	−0.014***
	(-1.34)	(-2.13)	(-1.57)	(-1.73)
UAV	0.009	0.014**	0.023*	0.019
	(0.57)	(2.16)	(5.46)	(1.05)
SMT	−0.133**	−0.112	−0.086	−0.092
	(-2.13)	(-0.94)	(-0.65)	(-1.16)
GR	0.063*	0.054**	0.043***	0.051*
	(3.29)	(2.17)	(1.71)	(4.64)
Constant	0.136	0.254	−0.144**	0.105
	(1.22)	(0.73)	(-2.04)	(1.56)
Y/I/C dummies	Yes	Yes	Yes	Yes
AR (2)	(0.235)	(0.328)	(0.344)	(0.278)
Hansen-test	(0.315)	(0.331)	(0.265)	(0.223)
Sargan-test	(0.285)	(0.257)	(0.322)	(0.362)

Note: This table shows the robust results. C/TA is the ratio of cash and cash balances to net assets; MtB is the market-to-book ratio; TD/TA is the ratio of total debts to total assets; ROE is the ratio of net income to total equities; DIV is dividend payout ratio; Ln (TA) is the natural logarithm of total assets; DR is the country risk index; IP is the annual score of protecting minority investors; UAV is the uncertainty avoidance value; SMT is the stock market turnover ratio; and GR is the global EPU index. Dummies include the years, industries, and countries. For coefficients in the models, t-statistics are reported in parentheses. The symbols *, **, and *** indicate statistical significance at the 1%, 5%, and 10% levels, respectively.

**Table 8:** Robustness test II.

Independent variables	Dependent variable: C/TA	Dependent variable: Log C/TA
	GMM in-Sys (Endogenous)	GMM in-Sys (Endogenous)
	Panel (A): Financial Risk Index (FRI)	
Lagged C/TA	0.345*	0.277*
	(4.67)	(3.55)
DR	−0.108***	−0.115*
	(-1.73)	(-3.56)
Firm, country, & global control variables	Yes	Yes
Y/I/C dummies	Yes	Yes
AR (2)	(0.342)	(0.337)
Hansen-test	(0.311)	(0.212)
Sargan-test	(0.254)	(0.356)
	Panel (B): Economic Risk Index (ERI)	
Lagged C/TA	0.296**	0.314*
	(2.13)	(5.36)
DR	0.084**	0.092*
	(2.14)	(5.32)
Firm, country, & global control variables	Yes	Yes
Y/I/C dummies	Yes	Yes
AR (2)	(0.326)	(0.344)
Hansen-test	(0.242)	(0.225)
Sargan-test	(0.317)	(0.231)
	Panel (C): Political Risk Index (PRI)	
Lagged C/TA	0.267*	0.227**
	(4.33)	(2.09)
DR	−0.319*	−0.277**
	(-4.33)	(-2.03)
Firm, country, & global control variables	Yes	Yes
Y/I/C dummies	Yes	Yes
AR (2)	(0.277)	(0.335)
Hansen-test	(0.369)	(0.416)
Sargan-test	(0.448)	(0.452)

Note: This table shows the robust results. The firm-level control variables include: MtB is the market-to-book ratio; TD/TA is the ratio of total debts to total assets; ROE is the ratio of net income to total equities; DIV is dividend payout ratio; Ln (TA) is the natural logarithm of total assets. For the country-level control variables, IP, UAV, and SMT are used. IP is the annual score of protecting minority investors; UAV is the uncertainty avoidance value; SMT is the stock market turnover ratio; GR is the global EPU index. Dummies include the years, industries, and countries. For coefficients in the models, t-statistics are reported in parentheses. The symbols *, **, and *** indicate statistical significance at the 1%, 5%, and 10% levels, respectively.

The findings of Eq. (1) in Table 7 indicate that the evaluated firm, country, and global-level factors significantly impacted Asian firms’ cash holdings. In addition, Asian firms stockpile greater amounts of cash as a result of rising DR and GR though the DR has a greater impact.

Table 8 displays the robust findings of Eq. (2). Overall, the results confirm the previous estimates in Table 5 that firms hold greater cash when financial and political risks increase while they react in the opposite direction when economic uncertainty increases. In line with the works by Refs. [14,18], our findings imply that firms have more precautionary motives for holding liquid assets when financial and political risks increase due to the possible upsurges in the cost of external funding and financial shortfalls. Also, it supports [13,14] works, which found that companies stockpile more cash and cash balances when countries have become economically stable.

Furthermore, we re-estimate the effects of DR and GR on cash holdings by categorizing economies based on new alternatives namely investor protection (IP), cultures (UAV), and financial market development (SMT) using the Sys-GMM approach. Table 9 reveals the findings. The robustness test results underscore that the countries' characteristics influence the extent of the effects of DR and GR on Asian firms’ cash holdings. Similar to the findings presented in Table 6, the estimation results corroborate that the impacts of DR and GR are more pronounced in environments having a high level of investor protection, a high uncertainty avoidance culture, and less financial market development. In such countries, the coefficient of DR is larger than GR, indicating that firms stockpile more cash and cash equivalents as a hedging instrument against increases in DR than GR. Following past works ([51,74]) which their findings showed that companies stockpile less cash in environments with strong legal settings, our results suggest that firms in strong investor protection settings due to holding mostly smaller cash have a relatively more precautionary incentive to stockpile larger cash as a shield against the increases of DR and GR. Notably, the precautionary motive of firms is strengthened in environments with well-protected shareholders as agency problems are less pronounced.

**Table 9:** Robustness test III.

Independent variables	Investor Protection (IP)		Culture (UAV)		Financial Markets (SMT)	
	Low protection	High protection	Low uncertainty	High uncertainty	Less development	High development
L.C/TA	0.336**	0.423*	0.344*	0.352**	0.451*	0.377*
	(2.03)	(5.24)	(4.54)	(2.12)	(4.22)	(4.57)
DR	−0.114*	−0.402*	−0.132**	−0.478*	−0.252*	−0.124**
	(-4.26)	(-3.36)	(-2.11)	(-4.55)	(-4.63)	(-2.16)
GR	0.011*	0.028**	0.016**	0.032***	0.054**	0.022***
	(3.25)	(2.41)	(2.17)	(1.73)	(2.09)	(1.68)
Firm & country Control variables	Yes	Yes	Yes	Yes	Yes	Yes
Y/I/C dummies	Yes	Yes	Yes	Yes	Yes	Yes
AR (2)	(0.344)	(0.224)	(0.335)	(0.421)	(0.214)	(0.367)
Hansen-test	(0.223)	(0.431)	(0.312)	(0.433)	(0.353)	(0.452)
Sargan-test	(0.356)	(0.326)	(0.263)	(0.252)	(0.266)	(0.202)

Note: This table shows the robust results. The firm-level control variables include: MtB is the market-to-book ratio; TD/TA is the ratio of total debts to total assets; ROE is the ratio of net income to total equities; DIV is dividend payout ratio; Ln (TA) is the natural logarithm of total assets. For the country-level control variables, IP, UAV, and SMT are used. IP is the annual score of protecting minority investors; UAV is the uncertainty avoidance value; SMT is the stock market turnover ratio; GR is the global EPU index. Dummies include the years, industries, and countries. For coefficients in the models, t-statistics are reported in parentheses. The symbols *, **, and *** indicate statistical significance at the 1%, 5%, and 10% levels, respectively. Dummies include the years, industries, and countries. The symbols *, **, and *** indicate statistical significance at 1%, 5%, and 10% levels, respectively.

Also, results support the prior works [54], implying that companies in environments with a higher uncertainty avoidance culture stockpile more cash as a way to safeguard against possible future country and global uncertainties. Moreover, the findings are consistent with the prior works [15], stressing that companies in environments with less developed financial markets have more transaction cost motive to hold more cash when DR and GR increase.

## 7 Conclusion

Although numerous empirical studies have probed the determinants of cash holdings in advanced and emerging economies, few works have mainly explored the impacts of country risk and global uncertainty on the cash holdings of Asian companies. Particularly, few studies attempted to test comprehensively the effects of country risk factors on Asian firms' cash holdings. Moreover, there is a gap in studying how countries’ characteristics influence the nexus between country risk, global uncertainty, and cash holdings. Therefore, this study aims to explore this nexus by choosing 989 listed Asian companies during the 2008–2020 period.

The findings reveal that a rise in country risk and global uncertainty stimulates the tendency of firms to hold more cash though the impact of country risk is more prominent in the investigated economies. Besides, the findings underscore that among the country risk factors, firms have more precautionary motives to stockpile more cash with increases in financial and political instability while they hold more cash with rises in economic stability. Likewise, the findings reveal that the impacts of country risk and global uncertainty on cash holdings are more prominent in environments characterized by a common-law legal origin, high uncertainty avoidance culture, and less financial market development. Moreover, the results highlight that the firm-specific factors including growth opportunity, leverage, profitability, dividend, and size have a significant role in describing Asian firms’ cash holdings policy. The findings of this study open another discussion in the literature that contributes to understanding the important role of country and global risks in explaining corporate cash-holding policies.

The findings have important policy recommendations. First, the findings suggest that regulators and policymakers in Asia should provide more stable environments for Asian companies so that they can reduce liquidity costs and manage associated costs more efficiently. Having a lower vulnerability to risk factors can reduce cash volatility and shortage, reducing the precautionary incentives for companies to hoard cash. Likewise, the reduction in financial and political risks allows Asian firms to hold less cash and instead exploit growth opportunities and increase their efficiency. This could be achievable by focusing on the relevant components of the financial risk (e.g., decreasing exchange rate volatility) and political risk (e.g., decreasing corruption, decreasing internal and external conflicts, and implementing the rule of law). Second, the results suggest that instead of increasing liquid assets, managers of Asian firms should diversify their asset compositions to hedge against the spillover impact of global uncertainty for utilizing investment opportunities and eventually raising the yields. Third, the results indicate that Asian firm executives should pay more attention to important firm-level factors namely growth opportunity, leverage, profitability, dividend, and size when deciding on cash holding policies.

It would be valuable for further work to examine the impact of country risk on cash holdings for other countries either individually or regionally. Besides, it would be interesting to probe the nexus between firm-level political risk, geopolitical risks, and cash holdings in Asian markets. In addition, it would be worthy to re-estimate this nexus by using the cash-to-sales ratio as another measurement of cash holdings. Likewise, it would be noteworthy to test the interaction impact of firm-level control variables and country and global risks in future studies. Moreover, it would be useful for further studies to extend the data range to consider the impact of COVID-19 on Asian firms’ cash holdings.

## Data availability statement

Data will be made available on request.

## Additional information

No additional information is available for this paper.

## CRediT authorship contribution statement

**Seyed Alireza Athari:** Writing – review & editing, Writing – original draft, Visualization, Validation, Supervision, Software, Resources, Project administration, Methodology, Investigation, Funding acquisition, Formal analysis, Data curation, Conceptualization. **Roland Cho Teneng:** Writing – original draft, Resources, Data curation. **Bülent Çetinkaya:** Writing – original draft, Visualization, Data curation. **Mahboubeh Bahreini:** Writing – original draft, Investigation, Data curation, Conceptualization.

## Declaration of competing interest

The authors declare that they have no known competing financial interests or personal relationships that could have appeared to influence the work reported in this paper.

## Figures and Tables

**Table A1:** Industry classifications

Industry classifications	Two-digit	Indonesia	Philippine	Malaysia	Singapore	Thailand	Taiwan	No. of sample	%Share of sample	%Share of total
Construction & materials	53, 57	18 (38)	11 (15)	78 (115)	55 (56)	30 (51)	38 (65)	230 (340)	67.64	23.26
Aerospace & defense	13, 17	─	1 (1)	1 (1)	4 (4)	─	4 (5)	10 (11)	90.90	1.01
General industrials	23, 27	6 (12)	3 (3)	19 (29)	9 (10)	7 (13)	6 (14)	50 (81)	61.72	5.06
Electronic & electrical equipment	33, 37	7 (10)	1 (1)	29 (40)	41 (42)	18 (22)	339 (403)	435 (518)	83.97	44.00
Industrial engineering	53,57	1 (5)	─	43 (52)	45 (59)	17 (31)	73 (119)	179 (266)	67.29	18.08
Support services	91, 93, 95, 97, 99	4 (8)	5 (5)	32 (39)	19 (33)	13 (26)	12 (30)	85 (141)	60.28	8.59
Total	─	36 (73)	21 (25)	202 (276)	173 (204)	85 (143)	472 (636)	989 (1357)	─	100
Sample (%)	─	49.31	84.00	73.18	84.80	59.44	74.21	72.88	─	─

Note: Table A1 shows the industry classifications based on two-digit ICB codes. For each industry classification, the number of samples (total) firms is reported. The last two columns report the percentage share of sample firms in each industry and the percentage share of each industry in total.

**Table A2:** Pearson correlation matrix

	MtB	TL/TA	ROA	DIV	Ln (TA)	DR	LO	UAI	SMC	GR	FRI	ERI	PRI	VIF
MtB	1.00													1.11
TL/TA	0.09*	1.00												1.12
ROA	0.11*	−0.22*	1.00											1.04
DIV	0.13*	−0.16*	0.11*	1.00										1.15
Ln (TA)	0.17*	0.15*	0.13*	0.19*	1.00									1.02
DR	−0.14*	−0.14*	0.08*	0.07*	0.09*	1.00								8.04
LO	0.12*	−0.07*	0.18*	−0.18*	0.04	−0.07*	1.00							1.09
UAI	0.08*	−0.14*	−0.09*	0.03	0.02	−0.11*	0.04	1.00						1.03
SMC	0.18*	0.22*	0.12*	−0.05	0.08*	0.15*	−0.03	0.05	1.00					1.14
GR	−0.11*	−0.09*	0.16*	0.12*	0.11*	0.23*	−0.05	0.12*	0.13*	1.00				1.05
FRI	−0.08*	−0.15*	0.13*	0.16*	0.10*	0.74*	0.08*	0.03	0.11*	−0.08*	1.00			10.13
ERI	−0.14*	−0.08*	0.18*	0.09*	−0.14*	0.78*	0.11*	0.09*	0.07	0.17*	0.47*	1.00		15.08
PRI	−0.09*	−0.12*	0.11*	0.15*	−0.16*	0.86*	−0.04	0.11*	0.14*	−0.15*	0.63*	0.58*	1.00	16.16

Note: * is statistically significant at the 1% level.

## Footnotes

1 According to the ICRG risk classification, countries with scores (of 00.0–49.9) and (80.0–100) are placed at “very high risk” and “very low risk” levels, respectively. In addition, countries with scores (of 50.0–59.9), (60.0–69.9), and (70.0–79.9) are placed at “high risk,” “moderate risk,” and “low risk” levels, respectively.

2 See www.prsgroup.com.

3 https://www.prsgroup.com/wp-content/uploads/2012/11/icrgmethodology.pdf.

4 www.policyuncertainty.com.

5 www.twse.com.tw.

6 This study found similar results by using another dependent variable of “Industry-Adjusted Cash Holdings”, which findings are not reported for the sake of brevity.
